# N-Cadherin Expression Is Associated with Acquisition of EMT Phenotype and with Enhanced Invasion in Erlotinib-Resistant Lung Cancer Cell Lines

**DOI:** 10.1371/journal.pone.0057692

**Published:** 2013-03-08

**Authors:** Xiaoju Zhang, Guangzhi Liu, Yi Kang, Zhaogang Dong, Qiyu Qian, Xitao Ma

**Affiliations:** 1 Department of Respiratory Medicine, Henan Provincial People’s Hospital, Zhengzhou, Henan, China; 2 Department of Obstetrics and Gynecology, Henan Provincial People’s Hospital, Zhengzhou, Henan, China; 3 Department of Infective Disease, Henan Provincial People’s Hospital, Zhengzhou, Henan, China; 4 Department of Clinical Laboratory, Qilu Hospital, Shandong University, Jinan, Shandong, China; Ospedale Pediatrico Bambino Gesú, Italy

## Abstract

**Background:**

The epidermal growth-factor receptor tyrosine kinase inhibitors have been effective in non-small cell lung cancer patients. However, acquired resistance eventually develops in most patients despite an initial positive response. Emerging evidence suggests that there is a molecular connection between acquired resistance and the epithelial–mesenchymal transition (EMT). N-cadherin is involved in the EMT and in the metastasis of cancer cells. Here, we analyzed N-cadherin expression and function in erlotinib-resistant lung cancer cell lines.

**Methods:**

H1650 cell lines were used to establish the subline resistant to erlotinib(H1650ER). Then, induction of the EMT was analyzed using immunostaining and western blots in H1650ER cells. N-cadherin expression in the resistant cells was examined using FACS and western blot. In addition, an invasion assay was performed to characterize the resistant cells. The effects of N-cadherin on cell proliferation and invasion were analyzed. The association of N-cadherin expression with the EMT phenotype was investigated using immunohistochemical analysis of 13 archived, lung adenocarcinoma tissues, before and after treatment with erlotinib.

**Results:**

In H1650ER cells, N-cadherin expression was upregulated, paralleled by the reduced expression of E-cadherin. The marked histological change and the development of a spindle-like morphology suggest that H1650ER cells underwent an EMT, accompanied by a decrease in E-cadherin and an increase in vimentin. A change in the EMT status between pre-and post-treatment was observed in 11 out of 13 cases (79%). In biopsies of resistant cancers, N-cadherin expression was increased in 10 out of 13 cases. Induction of the EMT was consistent with aggressive characteristics. Inhibition of N-cadherin expression by siRNA was tested to reduce proliferation and invasion of H1650ER cells *in vitro*.

**Conclusions:**

Our data provide evidence that induction of the EMT contributes to the acquired resistance to EGFR-TKIs in lung cancer. It suggests that N-cadherin is a potential molecular target in the treatment of NSCLC.

## Introduction

Lung cancer is the most common cause of death due to malignant carcinomas, and the use of traditional chemotherapeutic drugs against lung cancers are only modestly effective [1]–[3]. Recent advances using tyrosine kinase inhibitors that specifically block the epidermal growth factor receptor (EGFR) have provided a marked benefit to subsets of patients whose tumors harbor specific genetic abnormalities [4]–[7]. These drugs have led to impressive improvements in progression-free survival (PFS) compared to chemotherapy. However, drug resistance eventually emerges through various mechanisms [8]–[12]. This acquired resistance has been attributed to two main mechanisms. One suggested mechanism is the emergence of a malignant clone with a secondary mutation in the EGFR kinase domain (T790M), which abrogates the inhibitory activity of the TKIs [13]–[14]. Another 15 to 20% undergo amplification of the MET receptor tyrosine kinase, which activates downstream intracellular signaling independently from the EGFR [15]–[16]. Additionally, emerging evidence suggests that the acquisition of the EMT phenotype is related to the acquired resistance to EGFR tyrosine kinase inhibitors (TKIs) [17]–[20]. The EMT is characterized by the combined loss of epithelial cell junction proteins, such as E-cadherin, and the gain of mesenchymal markers, such as vimentin. In the EMT process, epithelial cells lose their features, gain mesenchymal properties, and become motile and invasive [21].

N-cadherin, a mesenchymal cadherin associated with the EMT, is crucial in cancer progression, with respect to both metastasis and to chemotherapy resistance. E-cadherin and N-cadherin share many structural and functional features. Both molecules establish calcium-dependent homophilic cell-cell adhesion with their extracellular domains and are connected with catenins at their intracellular domains [22]. One feature of the EMT is the reduction of cell–cell adhesions, especially the reduction of E-cadherin which is critical to maintaining the phenotype.

This study evaluated whether induction of the EMT was related to the acquired resistance observed during erlotinib treatment and to determine the role of N-cadherin in those patients with NSCLC before and after erlotinib treatment.

## Materials and Methods

### Cell Lines

The human lung adenocarcinoma cell line H1650 was obtained from the Chinese Academy of Science (Shanghai, China) and was cultured in a humidified 5% CO2 incubator at 37°C, in a DMEM solution containing 10% FBS, penicillin and streptomycin. The erlotinib-resistant cell line (H1650ER) was established by exposing parental cells to increasing concentrations of erlotinib, up to 10 µM, for several months. The resistant cells were continuously maintained in a medium containing 10 µM of erlotinib prior to each experiment.

### Reagents and Antibodies

Mouse anti–E-cadherin antibody(sc-21791) and monoclonal anti-vimentin antibody(sc-53464), Mouse anti–β-actin antibody(sc-47778), Rabbit anti–N-cadherin antibody (sc-59987), fluorescein isothiocyanate-conjugated goat anti-mouse antibody(sc-53800), and PE-conjugated goat anti-mouse antibody (sc-53464) were obtained from Santa Cruz Biotechnology (Santa Cruz, CA). Akt(#9272), S473 p-Akt(#4060), EGFR(#2239), and p-42/44 MAPK(#4695) were all purchased from Cell Signaling Technology (Danvers, MA). EGFR [pY1068] (CB11007889) and Rabbit anti–Erk antibody(CB1241938) were purchased from Affinity BioReagents (Golden, CO.).

### Cell Proliferation Assay

Cell proliferation assays were measured using the Cell Counting Kit-8 assay (CCK-8; Dojindo Laboratories, Santa Clara,CA), which uses the bioreduction of 2-(2-methoxy-4-nitrophenyl)-3-(4-nitrophenyl)-5-(2,4-disulfophenyl)-2H tetrazolium sodium (WST-8) to orange-colored formazan to measure cell viability. Briefly, 100 µl of H1650 and H1650ER cells, at 2×10^4^/ml, were incubated with different concentrations of erlotinib in 96-well culture plates for 48 hours. Next, the culture medium in the 96-wells was replaced with fresh medium containing 5% CCK-8 reagent; after an hour, the absorbance at 450 nm was measured and the values were corrected by subtracting the absorbance of control wells that did not contain cells.

### Immunohistochemistry

Tissues from 13 cases of lung adenocarcinoma, from both before and after erlotinib treatment, were collected between January 2008 and July 2011 in Henan Provincal People’s Hospital, and embedded in paraffin. Tumor tissue slides were de-paraffinized and dehydrated using Slide Brite (Sasco Chemical Group, Inc.). Antigen retrieval was performed using 0.05 M Glycine-HCL buffer, pH 3.5, containing 0.01% (w/v) EDTA, at 95°C for 20 min and stained with an antibody against human E-cadherin, vimentin and N-cadherin, which are same as for the in vitro assays,. The evaluation of immunostaining of these sections was made blind to two trained pathologists who were unaware of the clinical background of the samples. The intensity of E-cadherin,vimentin and N-cadherin were scored and placed into four categories according to staining: 0 for 0%; 1 for 1–33%; 2 for 34–66%; and 3 for 67–100%. All of the 13 human tissues involved in our study were carried out according to the principles set out in the Declaration of Helsinki 1964 and all subsequent revisions. The study was approved by the Ethics Committee of Henan provincial people’s hospital, Written informed consent was obtained from all subjects.

### Small Interfering RNA (siRNA) Transfection

Small interfering RNA (siRNA) specific for N-cadherin was purchased from Invitrogen. The sequence used for the N-cadherin interfering RNAs (siRNA) was 5′-CUAACAGGGAGUCAUAUGGUGGAGC-TdT-3′. To minimize nonspecific effects of interfering RNAs, non-targeting control siRNA, also purchased from Invitrogen, was used as negative control. The siRNA transfection reagent (Invitrogen) was used according to the manufacturer’s instructions. The experiment was repeated at least three times, independently.

### Matrigel Invasion Assay

Cells were treated with N-cadherin-specific siRNAs for 48 hours to knockdown N-cadherin expression. Invasion was observed using transwell culture inserts with a 6.5 mm diameter and 8 µm pore filters (Greiner Bio-One SAS, Courtaboeuf, France). Then, 3×10^4^ cells in 100 µl RPMI medium, with 1% FCS were seeded in the upper compartment, and 600 µl RPMI, with 10% FCS were added to the lower chamber. Cells were allowed to migrate for 24 hours at 37°C. After removing cells on the upper side of the transwell, cells on the underside were stained with 0.1% crystal violet solution (Becton Dickinson) and were lysed with 10% acetic acid for quantification by densitometric measurement at 550 nm. Experiments were carried out in triplicate.

### Western Blot Analysis

Proteins from the cell lysates, which were prepared using RIPA buffer [50 mM Tris, 150 mM NaCl, 1% TritonX-100, 0.1% sodium dodecyl sulfate and 1% Nadeoxycholate (pH 7.4)] supplemented with protease inhibitors (1 mM phenylmethylsulfonyl fluoride, 10 µg/ml pepstatin A, 10 µg/ml aprotinin and 5 µg/ml leupeptin), were detected by SDS-PAGE and then electrotransferred to Immobilon membranes (Invitrogen); these membranes were subsequently probed with specific antibodies. The secondary antibodies used horseradish peroxidase-conjugated antibodies and the membrane was developed using an ECL kit (Thermo). This experiment was repeated at least three times, independently.

### Clonogenic Assay

The H1650 and H1650ER cells were treated with 0.05% trypsin and plated in 6-well plates at 1×10^4^ cells per well in 0.35% agarose in DMEM medium layered on top of 0.7% agarose in the same medium. Colony formation was observed after 2–3 weeks in culture. Following staining with 0.005% crystal violet, colonies were counted under a microscope. Experiments were carried out in triplicate. The number of cells were counted and averaged to determine the cloning efficiency.

### Fluorescent Immunocytochemistry

Cells grown on coverslips were washed three times with PBS. They were fixed for 10 min with cold methanol and washed three times with PBS. The coverslips were then blocked in a solution of PBS, 10% bovine serum, and 0.1% Tween 20 for 1 h. The cells were incubated with primary antibody to either E-cadherin or to vimentin at 2 µg/ml for 1 h, then the cells were washed twice with PBS, and incubated with secondary antibodies diluted in PBS, 5 or 10% bovine serum, and 0.1% Tween 20. After incubating on coverslips for 1 h at 37°C the coverslips were washed as described above and mounted on slides in medium containing DAPI.

### Flow Cytometry Analysis

Cancer cells were dissociated using 0.05% trypsin; they were washed with PBS and 100 µl aliquots of 10^5^ cells in PBS were placed in 96-well V-bottom plates and incubated at RT for 1 h with IgG. Cells were washed twice with PBS. Following the washes with PBS, bound antibody was detected using goat anti-Human-PE or goat anti-Human-CY3, and analyzed by FACS (LSRII, Becton Dickinson).

### Statistical Analysis

The SPSS 13.0 statistical package (SPSS Inc., Chicago, IL) was used for data analyses. The significance of the difference among the covariates was determined by two-tailed v2 test. P values less than 0.05 were considered statistically significant.

## Results

### 1. The Acquisition of Erlotinib Resistance in H1650 Cell Lines Exhibited Variable Signaling Pathway Activation

**Figure 1:**
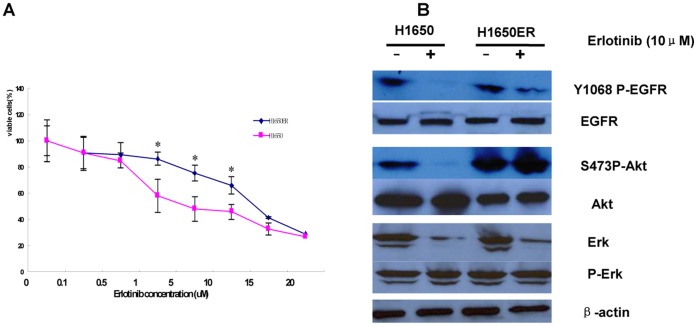
Generation of erlotinib-resistant H1650 cancer cells and variable signaling pathway activation. (A) Erlotinib-resistant cells (H1650ER) were established by long-term exposure to increasing concentrations of erlotinib. H1650 parental cells and H1650ER were seeded in 96-well plates at a density of 103 cells in 100 µL per well, cultured overnight, and treated with different concentrations of erlotinib for 72 h in a humidified incubator at 37°C with 5% CO2. The surviving fractions were determined by CCK-8 assay. Absorbance was measured at 450 nm. All experiments were performed in triplicate and S.D.s were calculated. *, P<0.01. (B) H1650 parental and H1650ER cells were cultured for 3 days with or without erlotinib treatment (10 µmol/L). Whole-cell lysates were prepared and analyzed by Western blot with the indicated antibodies.

The erlotinib-resistant lung cancer cell line H1650 (H1650ER), originally derived from the parental H1650 cell line, demonstrated an increased dependency on EGFR signaling for growth, as described previously [23]. By culturing this cell line in the presence of a constant high concentration of erlotinib over a period of several months, we have been able to isolate cell lines capable of growing at erlotinib concentrations up to 10 µM. Slow increases in growth occurred, indicating that a cell line resistant to erlotinib had been developed. The growth curves are presented in Fig. 1A.

Subsequently, we examined the mechanism of resistance to the EGFR-TKI erlotinib. Using Western blots, we probed lysates from both parental H1650 and H1650ER cells treated for 6 hours with erlotinib. Erlotinib treatment effectively blocked EGFR and Akt phosphorylation in the parental H1650 cells but not in the H1650ER cells. In these resistant cells, P-Akt was up-regulated and was evaluated as a candidate for involvement in the mechanism of acquired resistance. In contrast to the persistent Akt phosphorylation, Erk phosphorylation was down-regulated by erlotinib treatment in the resistant cells. These results suggested that the erlotinib-resistant cells adopted a new mechanism for activating the PI3K/Akt pathway (Fig. 1B).

### 2. N-cadherin Overexpression Induces Epithelial–mesenchymal Transition (EMT) in Erlotinib-resistant H1650ER Cells

**Figure 2:**
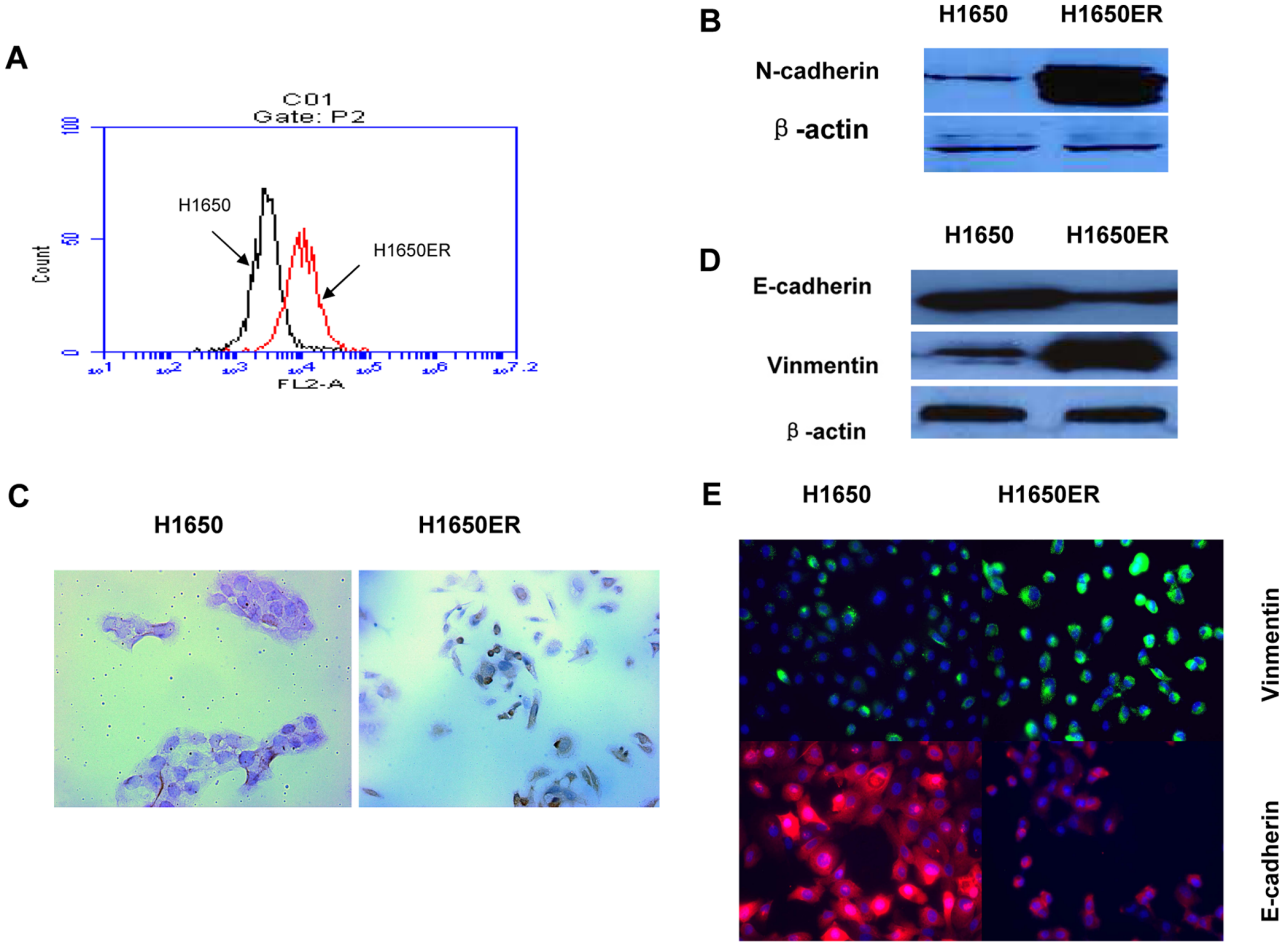
Up-regulation of N-cadherin expression in the H1650ER cells is accompanied by the induction of an EMT. (A) The upregulated expression of N-cadherin was detected by FACS analysis. (B) Cell lysates from H1650 and H1650ER cells were detected by Western blot; the lysates were also assessed for the level of N-cadherin protein. (C) The H1650 parental cells and the resistant H1650ER cells were assessed for morphological changes that are consistent with an EMT. Scale bars: 25 µm. (D) Reduced expression of E-cadherin and increased expression of vimentin were detected using Western blot. (E) Immunofluorescence staining was performed to validate the changes of the marker proteins from either the epithelial or the mesenchymal phenotypes. Scale bars: 25 µm.

To identify markers of erlotinib resistance, we compared gene expression in paired parental and resistant cell lines. N-cadherin expression was highly elevated in H1650ER cell lines, which we confirmed by FACS and western blot. (Fig. 2A, B). Next, morphological differences between the parental H1650 and the resistant H1650ER cell lines were readily observed (Fig. 2C). At the molecular level, these striking morphological features were associated with an increased expression of the mesenchymal protein vimentin and a decreased expression of the epithelial marker E-cadherin (Fig. 2D, E). Together, these findings indicate that the acquisition of erlotinib resistance induced molecular changes that were consistent with the EMT.

### 3. Up-modulation of N-cadherin Expression and the EMT were Found in Biopsies of Resistant Cancers

To identify the expression of N-cadherin and EMT biomarkers in EGFR-mutant NSCLC tissue samples, we performed biopsies on patients before erlotinib treatment and at the time that drug resistance was acquired. Thirteen patients had tumor tissue available both before and after TKI treatment. The characteristics of the 13 patients are shown in Table 1. Immunohistochemical staining of tumor specimens was used to analyze the protein expression of the epithelial marker E-cadherin and the mesenchymal marker vimentin. The epithelial markers were detected in nearly all biopsy specimens from NSCLC patients prior to erlotinib treatment, whereas mesenchymal markers were detected in 2 out of 13 tumor tissues. In resistant cancers, the expression of E-cadherin was decreased in 5 out of 13 biopsies. Moreover, in 11 (84.6%)out of 13 tumors, vimentin expression increased after erlotinib resistance was acquired; thus, a total of 11 specimens showed signs of an EMT in response to the induction of drug resistance.

**Table 1:** Summary of thirteen patients acquired resistance to erlotinib.

Case	Age	Sex	Genotype	Histology	Stage	Time on erlotinib*	Response^#^
1	59	Female	Exon 19 del	Adeno	IIIB	6months	PR
2	67	Female	L858R	Adeno	IIIA	12 months	PR
3	44	Female	L858R	Adeno	IIIB	13 months	PR
4	49	Female	L858R	Adeno	IIIB	7 months	PR
5	72	Male	Exon 19 del	Adeno	IIB	17 months	PR
6	72	Male	L858R	Adeno	IIIA	15 months	PR
7	61	Female	Exon 19 del	Adeno	IIIB	21 months	PR
8	62	Female	Exon 19 del	Adeno	IIIB	19 months	CR
9	68	Female	Exon 19 del	Adeno	IIIB	8 months	SD
10	57	Female	Exon 19 del	Adeno	IIIB	6 months	PR
11	59	Female	L858R	Adeno	IIIA	16 months	PR
12	64	Female	Exon 19 del	Adeno	IIIB	16 months	PR
13	65	Female	L858R	Adeno	IIIB	18 months	PR

Time on erlotinib*, Time to progression after erlotinib therapy; Response#, Response to erlotinib; del,deletion; Adeno, adenocarcinoma. CR: Complete response; PR: Partial response; SD: Stable disease; PD: Progressive disease.

**Figure 3:**
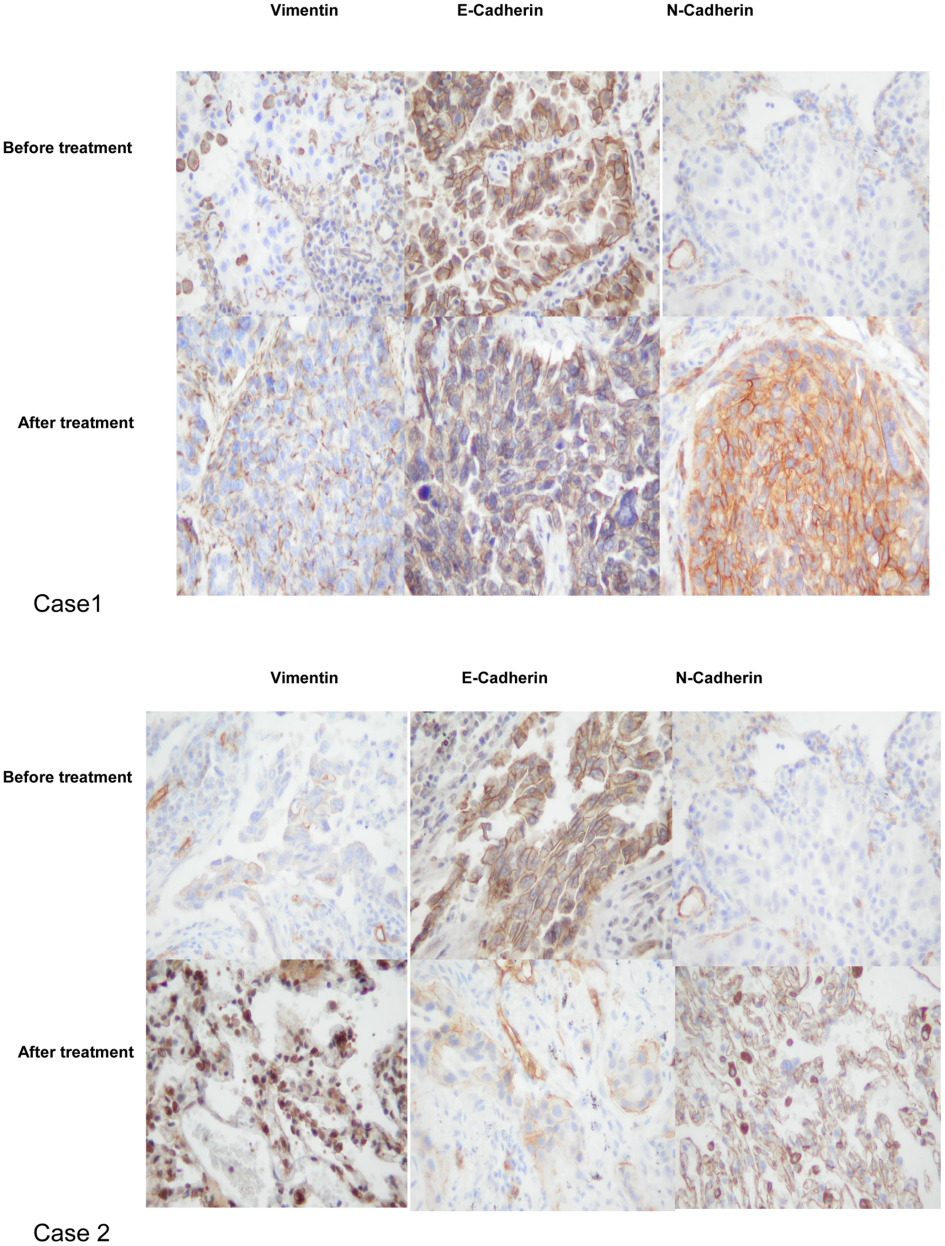
Up-modulation of N-cadherin expression and the EMT were found in biopsies of resistant cancers. (A) The loss of E-cadherin and an increase in both vimentin and N-cadherin expression were observed in these tumors (lower panel), compared with their corresponding primary tumors (upper panel). Scale bars:100 µm. Magnification, ×200.

Of the total cases, 12 out of 13 cases were negative for N-cadherin with the exception of one adenocarcinoma case that had focal N-cadherin expression before erlotinib treatment. On the other hand, the expression of N-cadherin was increased in 10 out of 13 biopsies in resistant cancers. Immunohistochemical expression were summarized in Table 2. Representative immunohistochemical stainings are shown in Figure 3A.

**Table 2:** N-cadherin, E-cadherin and Vimentin expression in tumor tissues.

	Marker Expression(pre-treatment)			Marker Expression(drug resistant)		
#	E-cadherin	Vimentin	N-cadherin	E-cadherin	Vimentin	N-cadherin
Case1	3	0	0	2	3	3
Case2	3	0	0	3	2	3
Case3	3	0	0	3	1	0
Case4	2	2	1	1	3	3
Case5	2	0	0	2	1	0
Case6	2	0	0	2	1	2
Case7	3	0	0	1	0	1
Case8	3	0	0	3	2	2
Case9	2	0	0	2	3	3
Case10	2	1	0	1	3	2
Case11	2	0	0	1	2	2
Case12	2	0	0	2	0	2
Case13	3	0	0	3	1	0

### 4. The Capability of Growth and Invasion was Increased in the H1650ER Cells

**Figure 4:**
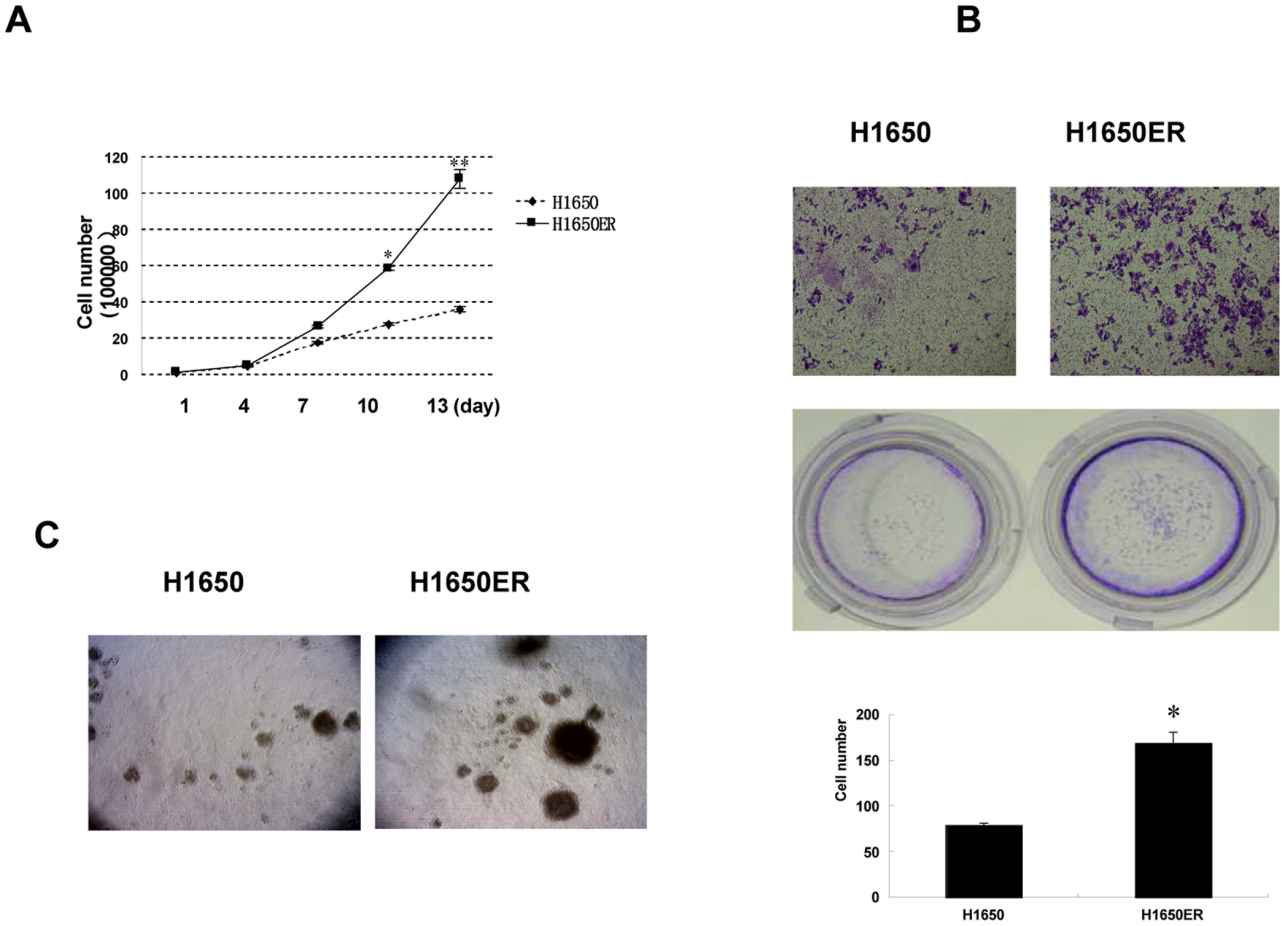
The capability of growth and invasion was increased in the H1650ER cells. (A) Growth curves of H1650, H1650ER cells and all other cells were performed in routine culture medium. Values show the mean cell number ± S.D. of triplicate wells at each time-point and represent three individual experiments. Error bars show SD; *, P<0.01; **, P<0.001. (B) Parental cells and H1650ER cells were seeded onto Matrigel-coated polycarbonate filters to analyze their invasive potentials. Scale bars: 25 µm. The results were obtained from three experiments, and the bars represent S.D.s; *, P<0.01. (C) Parental cells and H1650ER cell lines were also cultured in soft agar to test their ability to form colonies after 15 days of incubation. Original magnification,×10. The results were obtained from three experiments.

Induction of the EMT was consistent with aggressive characteristics, such as increased clonogenic growth, cell motility and invasion. To further characterize H1650ER cells, we compared the growth of parental and resistant cell lines using a cell-counting assay; the percentage of viable cells was calculated relative to the untreated controls (Fig. 4A). We also performed a Matrigel-coated chamber assay that showed increased cell invasion of H1650ER cells compared to parental cells (Fig. 4B). Moreover, we found that H1650ER cells acquired a more tumorigenic phenotype, as documented by increased clonogenic growth (Fig. 4C).

### 5. Down-modulation of N-cadherin Expression in the H1650ER Cells Resulted in Decreased Invasion

**Figure 5:**
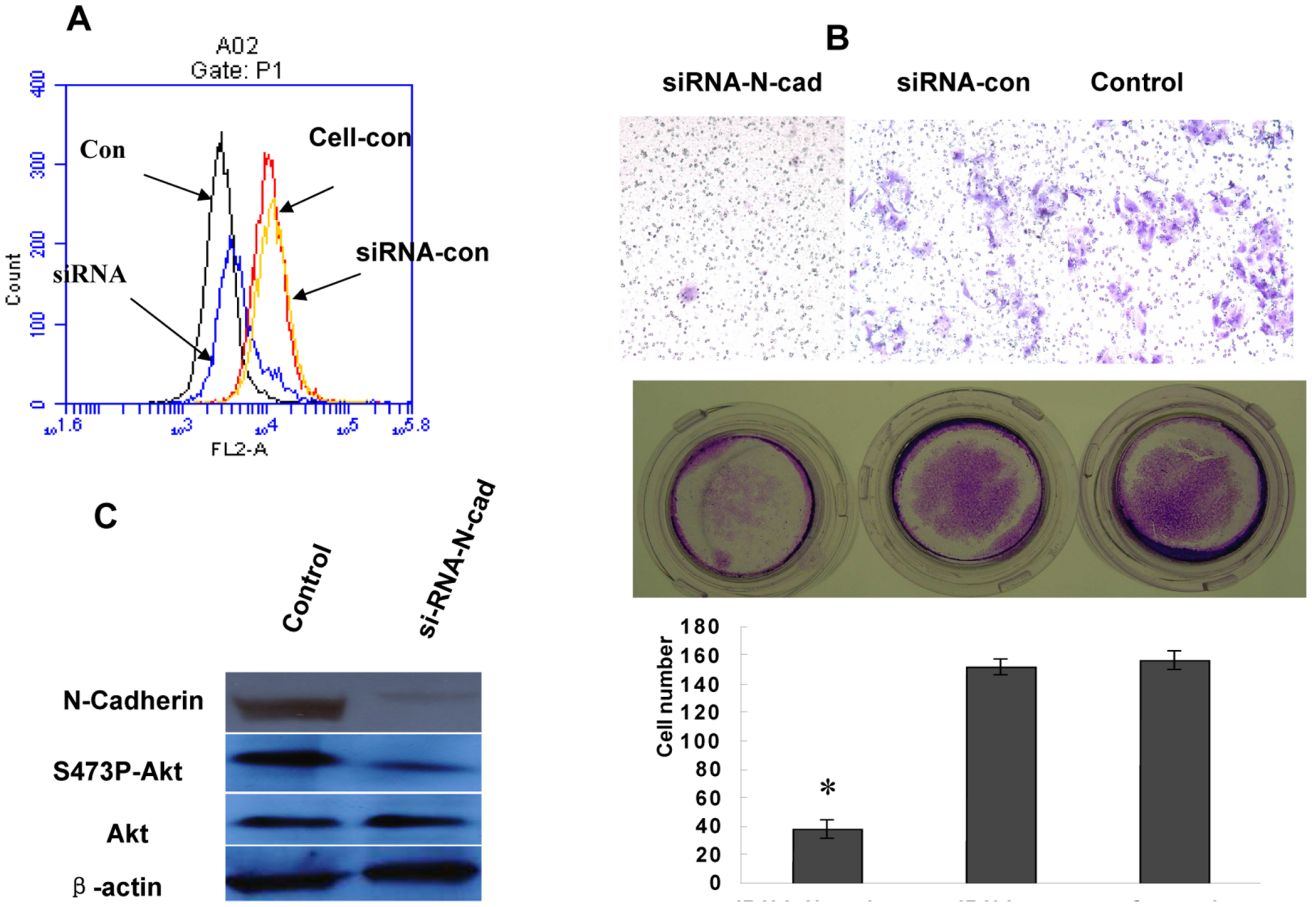
Downmodulation of N-cadherin expression and invasion abilities of H1650ER cell line. (A) N-cadherin expression in the H1650ER cells transfected with siRNAs targeting N-cadherin after 48 hours. Protein expression measured by flow cytometry, one representative experiment. (B) The ability of invasion evaluated after transfection for 48 hours, using a matrigel-coated chamber and 20% FCS as chemoattractant. Scale bars: 25 µm. (C) Changes in AKT and phospho-AKT kinase activity level upon N-cadherin siRNA silencing by Western blot.

The H1650ER cells expressing high levels of N-cadherin were down modulated by transient transfection with siRNAs. These efficient siRNAs produced significant reductions in N-cadherin protein (Fig. 5A) when compared to two control cell lines: the wild-type cell line and cell line transfected with lipofectamine. At the same time, N-cadherin silencing reduced AKT phosphorylation (Fig. 5B). The downregulation of N-cadherin expression was associated with reduced invasion abilities in the Matrigel-coated chamber when compared with wild-type cells or mock-transfected cells (Fig. 5C).

## Discussion

Here, we present evidence showing that acquired resistance to, EGFR-tyrosine kinase inhibitor, erlotinib in lung cancer is mediated by the epithelial–mesenchymal transition (EMT). In this study, we found that H1650 lung cancer cells developed resistance to erlotinib and underwent EMT phenotypic changes, which were consistent with decreased expression of the epithelial marker E-cadherin and increased expression of the mesenchymal markers vimentin. Moreover, in these clinical samples, the pretreatment cancers exhibited adenocarcinoma histology with preserved membranous staining for E-cadherin, while positive expression for vimentin was more frequently recognized in advanced, posttreatment cancers, indicating that some tumors underwent the EMT. Notably, our data indicate that EMT is associated with erlotinib resistance in NSCLC, as previously reported [24]–[25].

Aberrant expression of cadherins, or cadherin switching, is one of the key events in the EMT in certain types of cancer [26]–[27]. Inactivation of E-cadherin is an important event in tumor progression [28]–[29]. Abnormal activation of a cadherin, such as N-cadherin, would be a subsequent event which promotes angiogenesis, brain metastasis, and is associated with poor survival [30]–[31]. In our study, N-cadherin level is up-regulated in H1650ER cells. Immunohistochemistry also showed that N-cadherin expression was significantly upregulated in recurrent, posttreatment lung cancer specimens which gathered from 10 out of 13 patients. Moreover, siRNA-mediated knockdown of N-cadherin reduces the growth and invasion of H1650ER cells *in vitro*. The aberrant N-cadherin expressive status both *in vitro* and *in vivo* illuminates our hypothesis that, the crucial roles of N-cadherin reside not only in EMT, but also in tumor metastasis and erlotinib resistance. However, more expremental data are in need to verify our hypothesis. As not all the lung adenocarcinoma are sentitve to TKI, as only a few cell lines are sensitive to TKI, such as A549, PC9, HCC827, CALU-3, HCC4006 those harboring the acquired mutation in the EGFR gene [21], [32]–[35]. In the limited sensitive cell lines, the research on the relationship between N-cadherin and resistance is even less. Yamauchi M, et al [32] also found N-cadherin expression was significantly upregulated in gefitinib-resistant PC9/ZD cells harboring the acquired resistant mutation T790M in the EGFR gene, other cells expressing N-cadherin were found resistant to erlotinib (A549, H157, and H322) and that inhibition of N-cadherin expression using siRNA led to a significant decrease in viability in A549 and H322 cells.

Other researchers have reported that N-cadherin controls the motility and migration of cancer cells by suppressing Akt phosphorylation [36]–[37]. In agreement with the partial responsibility of p-Akt activation in invasion of some cancer cell lines expressing N-cadherin, we found that erlotinib blocked EGFR and ERK phosphorylation but not Akt phosphorylation in the erlotinib-resistant H1650ER cells. Persistent Akt phosphorylation in erlotinib-resistant lung cancer cells suggest that these cells adopt an alternative mechanism for activating PI3K/Akt to survive [38]–[39]. We propose that Akt activation might be associated with N-cadherin up-regulation, which was supported by our results that persistent Akt phosphorylation in erlotinib-resistant lung cancer cells could be overcome by an N-cadherin inhibitor. This seems to be consistent with the current literature. Tanaka H et al. show that N-cadherin silencing reduces AKT phosphorylation, whereas N-cadherin overexpression increases AKT activity in prostate cancer cells [40]; Similarly, Wallerand H et al. show that N-cadherin expression is associated with Akt activation and high invasiveness in human bladder cancer cell lines [41]. So it appears that N-cadherin is not only a biomarker of TKI resistance after exposure to EGFR inhibitors, but also a functioning molecule.

In summary, our study provides a further insight into the mechanisms involved in NSCLC and TKI resistance, revealing that the upregulation of N-cadherin in H1650 ER cells leads to increased tumor cell migration, invasion and tumorigenic potential. Our results also suggest that the maintenance of the EMT phenotype in H1650ER cells may be related to the sustained expression of N-cadherin. Therefore, N-cadherin may serve as a promising new target for the treatment of cancers with acquired resistance to EGFR TKIs. Because N-cadherin is expressed on the cell surface, we also ponder whether therapeutic targets using N-cadherin–specific monoclonal antibodies would have efficacy in those cancer cells with acquired resistance to EGFR tyrosine kinase.
